# Deciphering the Shifts in Microbial Community Diversity From Material Pretreatment to Saccharification Process of *Fuyu*-Flavor *Baijiu*

**DOI:** 10.3389/fmicb.2021.705967

**Published:** 2021-08-20

**Authors:** Jiamu Kang, Yunan Hu, Ziyuan Ding, Li Ye, Haoran Li, Jun Cheng, Lin Fan, Hu Zhao, Beizhong Han, Xiaowei Zheng

**Affiliations:** ^1^College of Food Science and Nutritional Engineering, China Agricultural University, Beijing, China; ^2^Nutrition & Health Research Institute, COFCO Corporation, Beijing, China; ^3^Beijing Key Laboratory of Nutrition, Health and Food Safety, Beijing, China; ^4^Jiugui Liquor Co., Ltd., Hunan, China

**Keywords:** saccharification, raw materials, pretreatment, microbiota, SourceTracker, *Fuyu*-flavor *Baijiu*

## Abstract

The microbiota of the pretreatment phase is crucial to the assembly of the microbial community in the saccharification of *fuyu*-flavor *baijiu*. This study investigates the shifts in microbial community diversity from the pretreatment of raw materials to the end of saccharification. High-throughput sequencing reveals that *Lactobacillus*, *Weissella*, and *Bacillus* in the bacterial community and *Rhizopus*, *Candida*, *Pichia*, and *Aspergillus* in the fungal community are predominant during raw material pretreatment and saccharification processes. Also, 11 bacterial genera, including *Bacillus*, *Lactobacillus*, *Leuconostoc*, *Weissella*, *Lactococcus*, and *Acetobacter*, and eight yeast genera, including *Candida*, *Pichia*, *Saccharomyces*, and *Wickerhamomyces*, were isolated from the initial saccharification stage by culture-dependent approaches. Sourcetracker analysis indicates that the cooling grains and rice husks were the main contributors to the bacterial community composition of the saccharification process, and *Qu* was the main contributor to the shaping of the fungal community structure during the saccharification process. Abundance variation of the predictive functional profiles of microbial communities encoding for key enzymes involved in pyruvate metabolism, starch and sucrose metabolism, and glycolysis/gluconeogenesis during the pretreatment and saccharification phases were inferred by PICRUSt2 analysis. The results of this study will be utilized to produce consistently high-quality *fuyu*-flavor *baijiu* via better controlling the shaping of microbial community structures during the pretreatment and fermentation processes.

## Introduction

*Baijiu* (Chinese liquor) is one of the oldest distillates globally, and it has various flavor types, most of which are produced by traditional manufacturing techniques in different regions of China (Zheng and Han, 2016; Liu and Sun, 2018). *Fuyu*-flavor *baijiu*, one of the famous mixed-flavor *baijiu* produced in the *Xiangxi* region, Hunan, China, is popular among consumers because of its fragrant aroma and sweet, mellow, and harmonious taste (Wu and Fan, 2009). The *fuyu*-flavor *baijiu* manufacturing process includes raw material pretreatment, starter preparation, solid-state saccharification, pit fermentation, distillation, aging, and blending. Traditionally, the manufacturing method uses a mixture of sorghum, rice, glutinous rice, and maize as raw materials and performs a preliminary solid-saccharification process after a series of raw material pretreatment processes. Then, the saccharified grains are mixed with *Daqu* starter to carry out fermentation in the pit. The solid-saccharification process enriches natural microbiota, contributing to the accumulation of unique and abundant flavor compounds (Wu and Fan, 2009; Zheng and Han, 2016).

Spontaneous saccharification is a complex biochemical process involving microbiota, including bacteria, yeasts, and filamentous fungi, resulting in high-quality properties of products. Raw materials are a key factor for *baijiu* flavor profiles, and the microbial community composition is highly correlated with the accumulation of flavor substances (Liu et al., 2019). The environment is regarded as an important contributor to the shaping of microbial community diversity during *baijiu* fermentation (Wang X. et al., 2018). The raw material pretreatment process of *fuyu*-flavor *baijiu* involves the mixing, soaking, cooking, and cooling of grains and then the addition of *Xiaoqu* (a *Rhizopus xiaoqu*, *Qu*) and rice husks (RH) to the pretreated grains. The entire pretreatment process is exposed to an open operation environment, and unsterilized cold water is used in the soaking and cooking processes. *Lactobacillus* introduced into the sorghum from the environment during the cooling processing is an important source of *Lactobacillus* during light-flavor *baijiu* fermentation (Pang et al., 2020). Also, RH is an auxiliary material used in the saccharification process as a filler for fermentation to improve the contact interface between microbiota and grains in the fermentation process and accelerate microbe propagation (Fan et al., 2020). These operations gather the environmental microbiota; however, considerably less is known regarding the microbial community composition shifts from pretreatment to the saccharification process.

Generally, the culture-dependent method offers quantitative data on microorganism occurrence and is widely used to isolate pure cultures from fermented samples for experimental fermentation tests, but it underestimates microbial diversity (Zheng et al., 2012). In recent years, high-throughput sequencing has promoted tremendous advances in the microbial community structure analysis of various traditional fermented foods based on the advantages of no culture, high throughput, and fast detection speed (Pang et al., 2018; Liu et al., 2020; Rizo et al., 2020). Hence, this study aimed to explain the shifts in microbial community structures and diversity in the pretreatment to saccharification stage via Illumina MiSeq PE300 sequencing. Predictive gene functionality of the bacterial and fungal community, involving pyruvate metabolism, starch and sucrose metabolism, and glycolysis/gluconeogenesis, were also inferred via Phylogenetic Investigation of Communities by Reconstruction of Unobserved States 2 (PICRUSt2). A culture-dependent approach was used to analyze further the samples’ microbial composition at initial saccharification. We identified 30 bacteria strains and 30 yeast strains randomly isolated from saccharified grains. These results create a basis for further research regarding the strains naturally occurring during saccharification having excellent fermentation and flavor-producing properties.

## Materials and Methods

### Experimental Design and Sample Collection

**FIGURE 1:**
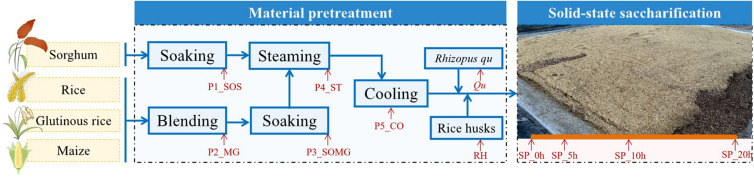
Diagram of the raw material pretreatment and solid-state saccharification processes of *fuyu*-flavor *baijiu*.

Samples were collected in a *fuyu*-flavor *baijiu* distillery in Hunan Province, China. Figure 1 shows the 11 detailed sampling sites whose name was marked “red.” The samples were collected at the beginning of raw material pretreatment until the end of saccharification, at P1_SOS, P2_MG, P3_SOMG, P4_ST, P5_CO, *Qu*, RH, SP_0h, SP_5h, SP_10h, and SP_20h. Approximately 100 g of grains with different pretreatment levels, *Qu*, RH, and grains in different saccharification stages at each point were aseptically collected in triplicate. To reduce sample heterogeneity, three production processes of the same batch were randomly selected, and triplicate subsamples were collected from each chain. All collected samples were immediately placed in an icebox for transportation to our laboratory for further analysis.

### Metagenomic DNA Extraction

For microbial metagenomic DNA extraction, one third of each of the three parallel samples was mixed. Then, DNA was extracted from 10 g of each mixed sample using the E.Z.N.A.^®^ Soil DNA Kit (Omega, Norcross, GA) according to the manufacturer’s instructions. Concentration of DNA was then quantified using a spectrometer. Then, 1% agarose gel electrophoresis was used to evaluate the integrity and purity of the extracted DNA under ultraviolet light. All extracted DNA samples were stored at −80°C before further experiments.

### Amplicon Sequencing

For bacterial PCR amplification, we targeted the hypervariable regions V3-V4 of the 16S ribosomal RNA (rRNA) gene using the universal forward primer 341F (5′-CCTAYGGGRBGCASCAG-3′) and reverse primer 806R (5′-GGACTACNNGGGTATCTA AT-3′). For fungal PCR amplification, we targeted the internal transcribed spacer (ITS) region using forward primer (5′-GGAAGTAAAAGTCGTAACAAGG-3′) and reverse primer (5′-GCTGCGTTCTTCATCGATGC-3′). The PCR products were checked by electrophoresis on 1% agarose gel and a Qubit fluorescence quantifier. The amplicon library preparation procedure was performed by the Illumina TruSeq DNA Sample Preparation Guide, and the amplicon sequencing was carried out using the Illumina MiSeq PE300 system (Illumina, San Diego, CA) according to the manufacturer’s standard protocols.

### Sequencing Data Processing and Bioinformatics

The original paired-end reads from Miseq sequencing were merged via FLASH software (version 1.2.11) (Magoc and Salzberg, 2011). Trimmomatic software (version 0.33) was employed to carry out the quality filtering of the merged sequences (Bolger et al., 2014). Subsequently, UCHIME software (version 8.1) was employed to identify and remove chimera sequences. Operational taxonomic units (OTUs) were clustered from clean tags with 97% similarity using UPARSE software (version 10.0) (Edgar, 2013). Then, to achieve the microbial taxonomic annotation, the representative bacterial OTU sequences were aligned with the Silva databases (Release132^1^), and the representative fungal OTU sequences were aligned with Unite databases (Release 8.0^2^). Sequencing data are available at NCBI with Sequence Read Archive (SRA) accession: PRJNA705302.

Per the OTU cluster information, rarefaction curves and alpha diversity indices, including observed OTU number, Chao1 richness estimator, Ace richness estimator, Shannon diversity index, Simpson diversity index, Good’s coverage, and phylogenetic diversity (PD whole tree), were calculated with the QIIME2 program (Bolyen et al., 2019). The beta diversity was evaluated using the Bray–Curtis distance matrix and was further visualized via Origin Lab software (version 9.0). The SourceTracker program was used to predict the sources of microbial communities in grain samples at different saccharification stages based on the default parameters. The Interactive Tree of Life (iTol) was used to visualize and annotate phylogenetic trees. In addition, PICRUSt2 predicted functional profiling of the metagenome based on bacterial and fungal amplicon sequencing profiles (Douglas et al., 2020). The functional annotation of predicted features was carried out based on the Kyoto Encyclopedia of Genes and Genomes (KEGG) database. A raw OTU count data set was performed through the PICRUSt2 pipeline with default parameters. Then, Enzyme Commission (EC) numbers and metabolic process abundances in each given sample were imputed. The relative abundance of each individual EC term related to pyruvate metabolism, starch and sucrose metabolism, and glycolysis/gluconeogenesis was calculated and visualized.

### Isolation and Identification of Bacteria and Yeasts From the Initial Saccharification

Culturable bacteria and yeasts of the initial saccharification samples were analyzed by the culture-dependent method as described by Zheng et al. (2012). A total of 10 g of sample was transferred into sterile stomacher bags and homogenized with 0.85% (w/v) sterile NaCl solution (90 mL). An appropriate serial dilution was performed, and 100 μL of the diluted suspension was dispensed on different selective agar media. Lactic acid bacteria (LAB) were grown on De Man, Rogosa, and Sharpe agar (MRSA) with 500 μg/mL natamycin at 37°C for 48 h to achieve distinct LAB colonies. Thermoduric bacteria and Enterobacteriaceae were grown on Luria–Bertani agar (LBA) and violet red bile glucose agar (VRBGA), respectively, and were incubated at 37°C for 24 h. Yeasts were grown on potato dextrose agar (PDA) and incubated at 28°C for 48 h. Then, one to three colonies with different morphologies were randomly isolated from each plate for purification and identification. Pure cultures were stored at −80°C in glycerol (30% v/v) stocks. Total DNA of bacteria and yeast were extracted using a bacterial or fungal genomic DNA extraction kit, respectively (Tiangen, Beijing, China). The 16S rRNA gene of bacterial strains and the ITS gene of yeasts were amplified using the primers 27F/1492R and ITS1/ITS4, respectively (Dong et al., 2020). The resulting PCR products were purified, cloned, and sequenced using the Sanger sequencing platform (BGI Inc., China). The sequencing results were compared with the BLAST algorithm’s GenBank database (National Center for Biotechnology Information, United States). Nucleotide sequences of evaluated bacteria and yeasts have been deposited at GenBank with accession numbers MZ057699-MZ057728 and MZ089518-MZ089547, respectively.

## Results

### Characteristics of the Sequencing Results and Alpha Diversity

Metagenomic DNA extracted from pretreated and saccharified samples was detected via an amplicon sequencing tool, generating 586,131 total raw reads for 16S rRNA gene sequences with an average of 44,569 clean reads for the bacterial community. For the fungal community, a total of 1,640,536 raw ITS gene sequences were collected; after quality control, an average of 124,217 clean reads were obtained per sample. Based on a similarity threshold of 97%, the number of OTUs clustered from these reads and their diversity index are exhibited in Table 1. A total of 173 bacterial and 120 fungal OTUs were identified. Microbial OTUs were annotated further at the phylum and genus levels. All bacterial OTUs were assigned into 8 different phyla, 13 classes, 25 orders, 46 families, and 100 genera. Fungal OTUs were assigned into 3 phyla, 9 classes, 16 orders, 33 families, and 50 genera.

**TABLE 1:** Observed operational taxonomic units (OTUs), richness indices (ACE and Chao1), diversity indices (Simpson and Shannon), phylogenetic diversity (PD whole tree), and Good’s coverage of microbial community.

	**Samples**	**OTU**	**ACE**	**Chao1**	**Simpson**	**Shannon**	**PD whole tree**	**Coverage**
Bacterial community	P1_SOS	102	109.0323	111.4286	0.8085	3.1533	8.1128	0.9997
	P2_MG	124	130.0231	128.5833	0.7058	2.5683	9.0968	0.9998
	P3_SOMG	72	74.852	73.6667	0.5266	1.9845	6.5195	0.9999
	P4_ST	92	104.1773	113.8571	0.7167	2.3786	7.5156	0.9997
	P5_CO	101	107.5016	106.5	0.5112	2.1258	7.7153	0.9998
	*Qu*	115	116.3863	116	0.7189	2.9506	9.463	0.9999
	RH	92	100.1536	103.375	0.821	3.0425	7.1955	0.9996
	SP_0h	82	85.6321	84.1	0.7961	2.8368	7.5458	0.9998
	SP_5h	61	72.9297	68.8	0.5069	1.6933	5.6612	0.9997
	SP_10h	70	83.8043	80.1111	0.6263	2.0241	6.5261	0.9996
	SP_20h	98	107.5217	106.0769	0.5274	1.9752	8.3716	0.9995
Fungal community	P1_SOS	76	104.0427	86.1111	0.4792	1.7773	11.4923	0.9999
	P2_MG	101	103.6779	103.5	0.469	1.9906	16.377	1
	P3_SOMG	45	57.8549	56	0.0953	0.4364	8.7696	0.9999
	P4_ST	104	104	104	0.8763	3.9352	16.9568	1
	P5_CO	18	23.8787	19	0.2608	0.8991	5.4322	1
	*Qu*	16	73.2319	38.5	0.0011	0.0083	5.4795	0.9999
	RH	80	82.9827	81.6667	0.7376	2.5836	14.8141	1
	SP_0h	22	23.2597	22.125	0.0304	0.1464	7.4477	1
	SP_5h	48	53.6663	50.625	0.5134	1.4434	9.4758	0.9999
	SP_10h	31	41.3997	32.6667	0.2927	0.7387	8.8735	1
	SP_20h	15	30.7193	24.3333	0.0137	0.0622	5.0977	0.9999

The Shannon and Simpson indices reflect the community diversity and evenness for the alpha diversity indices, and the Chao1 and ACE estimator characterizes the community richness (Liu et al., 2020). Among these samples, the bacterial and fungal ACE and Chao1 indexes were higher in P2_MG and P1_SOS samples, indicating higher microbial community richness. Moreover, from the viewpoint of bacterial Shannon and Simpson indices, P1_SOS and RH had the higher community diversity although the samples in the saccharification stage from 5 to 20 h had lower bacterial diversity. P4_ST had the highest community diversity from the fungal diversity aspect, and both *Qu* and SP_20h had the lowest fungal diversity. The phylogenetic diversity (PD) index of the tree reflects the genetic relationship of species. The PD whole tree value of *Qu* in the bacterial community was 9.463, the biggest among all samples, indicating a complex genetic relationship. In contrast, the P4_ST had the most complex genetic relationship in the fungal community and the farthest evolutionary distance among all groups. Also, the Good’s coverage estimators of all samples were near 1.00, suggesting that the sequencing depth is enough to represent the studied microbial community.

### Changes in the Bacterial Community During Pretreatment and Saccharification Process

**FIGURE 2:**
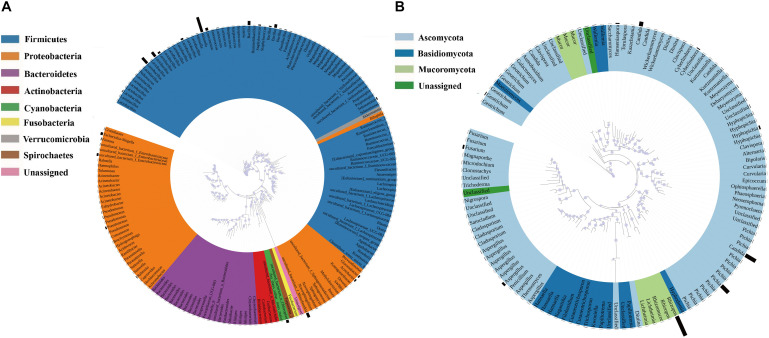
Phylogenetic tree of bacterial **(A)** and fungal OTUs **(B)** found in all samples. Color ranges identify phyla within the tree. Bars represent the relative abundance of each OTU in all samples.

**FIGURE 3:**
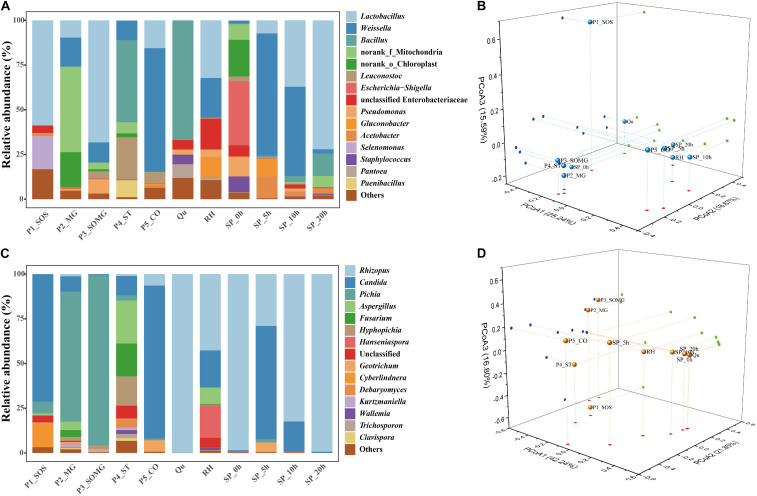
Relative abundance of bacterial **(A)** and fungal communities **(C)** in pretreated and saccharified samples. PCoA analysis of the bacterial **(B)** and fungal communities **(D)**, based on the Bray–Curtis distance.

Annotation of the samples’ generated bacterial OTUs is shown in Figures 2A, 3A. Firmicutes and Proteobacteria were the main bacterial phyla identified in all samples. At the genus level, the evolutionary relationship of 173 bacterial OTUs from all samples was constructed. *Lactobacillus*, *Weissella*, and *Bacillus* comprised the top three genera, and *Lactobacillus* was the most abundant genus (Figure 2A). In detail, the most dominant bacteria in the soaked sorghum sample was *Lactobacillus* with an abundance of 58.63%. In contrast, the dominant bacteria in the rice, glutinous rice, and maize mixture were norank_f_ Mitochondria (47.78%), norank_o_Chloroplast (19.65%), *Weissella* (16.29%), and *Lactobacillus* (9.65%). After the steeping of mixed grains, *Lactobacillus* (68.25%) remained the most abundant bacterial genus, followed by *Weissella* (11.30%).

*Bacillus* (45.91%) became the dominant bacterial genus in the cooked soaked grains due to their excellent heat resistance, and the relative abundance of *Lactobacillus* was reduced to 0.18%. After cooling, the main bacterial genera identified in the pretreated grains were *Weissella* (69.16 %) and *Lactobacillus* (15.58 %). *Qu* and RH were used as a starter and auxiliary material in the solid-state saccharification process, respectively. *Bacillus* was detected as the dominant bacterial genus in the *Qu*, followed by *Pantoea* (7.67%), *Staphylococcus* (5.38%), and unclassified Enterobacteriaceae (5.28%). *Lactobacillus* (32.25%), *Weissella* (22.13%), unclassified Enterobacteriaceae (17.18%), and *Gluconobacter* (11.21%) were preponderant genera in RH.

The saccharification process’s initial bacterial structure mainly included *Escherichia-Shigella*, norank_o_Chloroplast, *Pseudomonas*, *Staphylococcus*, norank_f_Mitochondria, unclassified Enterobacteriaceae, *Weissella*, *Bacteroides*, and *Lactobacillus*, comprising 35.91%, 20.57%, 11.07%, 8.76%, 8.74%, 6.29%, 1.43%, 0.19%, and 0.18% of the bacterial community, respectively. As the saccharification process progressed, LAB gradually became the dominant bacterial genera; in particular, *Lactobacillus* showed a linear increase from 0 to 20 h.

To further understand the composition of natural cultivable bacteria and collect functional strains for laboratory-scale research, culture-dependent isolation coupled with the 16S rRNA gene identification method were used to isolate and identify the bacteria. A total of 30 bacterial strains were randomly isolated from the samples of the initial saccharification stage. After 16S rRNA gene sequencing, these isolated strains were matched against GenBank of NCBI, and the detailed assignments can be found in Supplementary Table 1. As shown in Supplementary Table 1, all strains were classified into 11 bacterial genera, including *Bacillus* (*n* = 7), *Lactobacillus* (*n* = 6), *Leuconostoc* (*n* = 4), *Weissella* (*n* = 3), *Lactococcus* (*n* = 3), *Acetobacter* (*n* = 2), *Staphylococcus* (*n* = 1), *Paraburkholderia* (*n* = 1), *Acinetobacter* (*n* = 1), *Franconibacter* (*n* = 1), and *Pantoea* (*n* = 1).

A PCoA plot assessed the beta diversity of the bacterial community structure. The first three principal components (PCoA1, PCoA2, and PCoA3) explained more than 59% of the total variance (Figure 3B). P1_SOS and *Qu* samples were separate from all other samples. The bacterial community structures of P2_MG, P3_SOMG, and P4_ST samples in the pretreatment stage were relatively similar to the bacterial community during initial saccharification. The P5_CO and RH samples were closer to the bacterial composition of the saccharification process. These results indicated that the bacterial community structures in the pretreatment process were closely related to the saccharification process.

### Changes in the Fungal Community During Pretreatment and Saccharification Process

A phylogenetic tree of 120 fungal OTUs was constructed to perform the evolutionary relationship of fungi (Figure 2B), *Rhizopus*, *Candida*, *Pichia*, and *Aspergillus* comprise the top four fungal genera. The generated fungal OTU sequences were mainly ascribed to three phyla: Ascomycota, Mucoromycota, and Basidiomycota. At the genus level (Figure 3C), *Candida* (71.27%), *Cyberlindnera* (13.35%), and *Pichia* (6.52%) were the dominant fungal genera in the P1_SOS. In the P2_MG and P3_SOMG samples, *Pichia* was the most important fungi. *Aspergillus* (24.07%), *Fusarium* (18.32%), *Hyphopichia* (16.45%), and *Candida* (10.68%) became the dominant fungal genera after the soaked grains were cooked. After the steamed grains were cooled down, *Candida* was the main genus comprising 80% of the fungal community. *Rhizopus* was the fungal genus occupying 99.94% abundance in the *Qu* sample. The RH sample’s predominant genera were *Rhizopus*, *Candida*, *Hanseniaspora*, and *Aspergillus* with 42.71%, 20.73%, 18.15%, and 9.09% respective relative abundances. Due to the fermentation starter, the fungal community at the initial stage of saccharification was similar to the *Qu* sample. In the SP_5h sample, *Candida* was the dominant fungal genus, followed by *Rhizopus*. In the subsequent saccharification process, the relative abundance of *Candida* gradually decreased, and the *Rhizopus* gradually increased. By the end of saccharification, the proportion of *Rhizopus* reached 99.31%.

To further understand the natural yeast compositions and collect pure cultures for laboratory-scale fermentation, a total of 30 yeasts were randomly isolated from the samples of the initial saccharification stage by the culture-dependent method coupled with ITS gene identification. These isolated strains were matched against GenBank using BLAST analysis as shown in Supplementary Table 2. Isolated strains were identified as yeast belonging to eight genera, including *Candida* (*n* = 6), *Pichia* (*n* = 9), *Saccharomyces* (*n* = 5), *Wickerhamomyces* (*n* = 5), *Issatchenkia* (*n* = 2), *Debaryomyces* (*n* = 1), *Diutina* (*n* = 1), and *Hyphopichia* (*n* = 1).

As shown in Figure 3D, the first three principal components were constructed in the PCoA plot, explaining 42.24%, 21.85%, and 16.80% of the variance in the data set, respectively. SP_0h, SP_10h, SP_20h, and *Qu* samples were closer on the map, suggesting that *Qu* plays an essential role in shaping the fungal community during saccharification.

### The Microbial Association Between Pretreatment and the Saccharification Process

**FIGURE 4:**
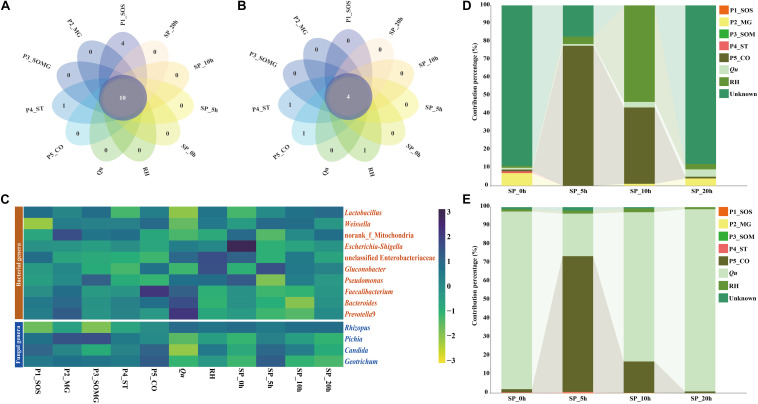
The petal diagram shows the unique and shared bacterial **(A)** and fungal genera **(B)** among different samples. The heat map shows the relative abundance of the microbial genera shared in all samples **(C)**. Sourcetracker results highlight the contribution percentages of the source bacterial **(D)** and fungal **(E)** community in the pretreatment stage to the saccharification stage.

Petal plots were constructed to investigate the similarities and differences between the pretreated and saccharified samples’ bacterial and fungal communities (Figure 4). As illustrated in Figures 4A,B, 10 bacterial genera and four fungal genera, which were most common to all the samples, were classified as core genera. Figure 4C shows the distribution of these common bacterial and fungal genera during the pretreatment and saccharification stages. Lactic acid bacteria was the dominant bacterial genus in the pretreatment stage of raw materials and the late saccharification stage. The dominance of *Rhizopus* in the fungal community mainly appeared after the adding of *Qu*. During the pretreatment processes of raw materials, the main fungal genera in all samples were mainly various yeasts. Moreover, for the bacterial community, except for the two samples P4_ST and *Qu*, the abundance of these common bacterial genera in the remaining samples accounted for most of the total 16S rRNA sequences (greater than 60% bacterial composition). The core fungal abundance in P3_SOMG, P5_CO, and *Qu* samples and the four samples collected from saccharification processes accounted for more than 95% of the total ITS sequences for the fungal community.

As shown in Figures 4D,E, a source tracker was employed to predict the sources of bacteria and fungi found in saccharified grains. For the bacterial community, 90.04% of the bacteria in the SP_0h sample was from an unknown source, 6.62% was P2_MG-derived, and the remaining pretreated samples’ contribution was less than 1%. In the SP_5h samples, P5_CO and RH samples contributed 76.49% and 4.21% of the bacteria, respectively. The samples P5_CO, RH, and *Qu* contributed 98.3% of the bacterial community in the sample at the 10^*th*^ hour of saccharification. In the sample of SP_20h, most of the bacteria did not originate from the pretreatment stage, and only 10.44% of the bacteria came from the three pretreatment samples of P2_MG, *Qu*, and RH. For fungal communities, *Qu* was the primary fungal source of both SP_0h (95.6%), SP_10h (80.2%), and SP_20h (97.9%) samples, whereas P5_CO was the main contributor to the fungal community of sample SP_5h. P5_CO sample contributed 73.0% of the fungal community to the SP_5h sample and 16.8% to the SP_10h sample.

### Changes in the Expression of Predicted Functional Enzymes During Pretreatment and the Saccharification Process

**FIGURE 5:**
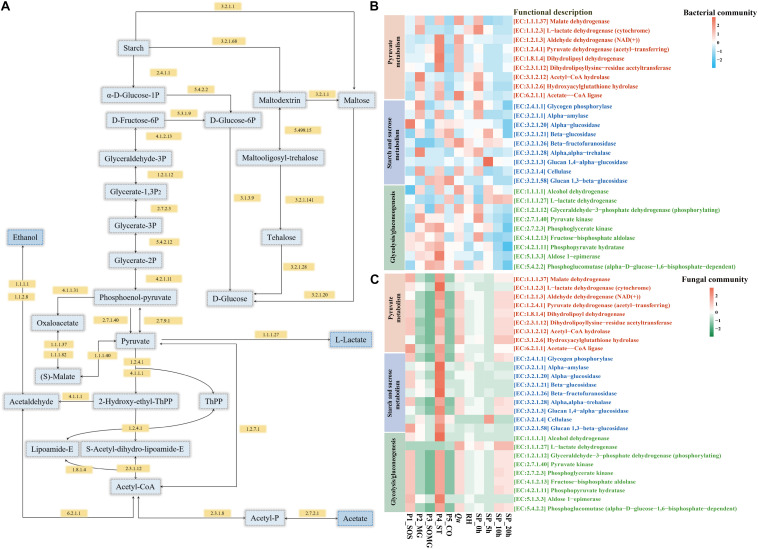
Schematic overview of the metabolic processes involving pyruvate metabolism, starch and sucrose metabolism, and glycolysis/gluconeogenesis **(A)**. Heat map shows changes in the relative abundance of annotated functional enzymes related to saccharification and the formation of flavor compounds [**(B)** bacterial community and **(C)** fungal community].

The PICRUSt2 platform contains an updated and more extensive database of gene families and reference genomes, which provides improved accuracy and flexibility for marker gene metagenome inference (Douglas et al., 2020). In this study, we used PICRUSt2 to predict the metagenome functions of the bacterial and fungal community during raw material pretreatment and saccharification processes. Figure 5 shows the changes in the enzyme-encoding genes of bacteria and fungi as annotated using KEGG regarding the categories of pyruvate metabolism, starch and sucrose metabolism, and glycolysis/gluconeogenesis. For pyruvate metabolism of the bacterial community, the relative abundance of core enzymes in the P4_ST, *Qu*, and SP_0h samples was higher. The functional enzymes involved in starch and sugar metabolism of the bacterial community gradually decreased in the middle and late stages of saccharification (Figure 5B). In contrast, the relative abundance of these key enzymes in the fungal community showed different changes. The enzymes of the fungal community regarding pyruvate metabolism in the P1_SOS, P4_ST, *Qu*, SP_10h, and SP_20h were relatively high than in other samples. Meanwhile, P4_ST contributed a higher abundance of functional enzymes in the fungal communities (Figure 5C). For these important enzymes presented in Figures 5A,C, the fungal community of samples collected from saccharification stage showed a high abundance of these enzymes. These findings suggested that bacteria and fungi contributed to the rich metabolic activities of the saccharified grains.

## Discussion

The pretreatment of raw material is an integral part of the fermentation processing of *baijiu* as it changes the property of the material and is beneficial to the growth and metabolism of microorganisms. The microbial community associated with alcoholic fermentation is related to the fermentation starters and the environmental microbiota during material pretreatment (Pang et al., 2020). In this study, we systematically investigated the changes in microbial composition and function from the raw material pretreatment to the saccharification processes of the *fuyu*-flavor *baijiu*. We analyzed the potential contribution of each operation of the pretreatment process to the shaping of the microbial community in the saccharification process. Many functional microorganisms exhibited in the saccharification process, such as LAB, *Bacillus*, and yeasts, might derive from environment, raw material, and unsterilized cold water. A series of pretreatment operations may display a positive impact on shaping the naturally sourced microbial communities. Thus, we used the culture-dependent method to isolate naturally sourced bacteria and yeasts from the initial saccharification samples. On the one hand, the acquisition of these strains assisted the high-throughput sequencing results to explain the microbial composition of saccharification at the species level. On the other hand, these results laid the foundation for the synthetic functional microbiota and the management of the saccharification process.

In this study, we found that the abundance of LAB in grains increased after soaking treatment. Pang et al. (2020) reported that *Lactobacillus* (84.6%) was the dominant bacterial genus after the steeping of sorghum for approximately 20 h, which is consistent with our results. Steeping of cereal-based raw materials can be practiced to increase bioactive components and reduce the content of some antinutritional components and fermentation with LAB, which can further accumulate functional bioactive components (Hassani et al., 2016). High-temperature favors thermotolerant and aerobic endospore-forming bacteria (Zheng et al., 2014). This explains why *Bacillus* members became the dominant bacteria in the P4_ST sample. Members of *Bacillus* genera were regarded as a constant factor in *baijiu* fermentation starter. They can contribute to the formation and accumulation of flavor compounds and functional enzyme activities (i.e., amylases and proteinases) needed to ferment cooked sorghum for alcoholic fermentation (Zheng et al., 2011). In the solid-state saccharification process of rice-flavor *baijiu*, LAB species are potentially responsible for the high amount of lactic acid in the sample after saccharification (Yin et al., 2020). Pang et al. (2018) reported that *Lactobacillus* was positively correlated with essential esters in *baijiu*. These may be important reasons for the accumulation of flavor compounds in the saccharification process. The results of culture-dependent analyses show 16 LAB strains, including *Lactobacillus hilgardii*, *Lactobacillus fermentum*, *Lactobacillus paracasei*, *Lactobacillus plantarum*, *Lactococcus lactis*, *Lactococcus lactis* subsp. *Hordniae*, *Lactococcus taiwanensis*, *Leuconostoc lactis*, *Leuconostoc pseudomesenteroides*, *Weissella confusa*, and *Weissella cibaria*, were found in the samples collected from the initial saccharification stage. In contrast, LAB populations were not dominant in the bacterial diversity as shown by the high-throughput perspective of the initial saccharification sample. The reason could be that these LAB populations were not picked up by high-throughput sequencing because of their low numbers but could be enriched by culture-dependent approaches (Zhadyra et al., 2021). In addition, the low abundance of *Bacillus* during saccharification may be due to the lower oxygen concentration, and higher alcohol content prevented the growth of *Bacillus* (Ren et al., 2019).

*Rhizopus* is a fungal genus widely used as a bioconversion organism in solid-state fermentation. Several characteristics, including wide growth temperature interval, wide growth and survival pH range, board fermentative substrates, and the range of by-products produced, make this genus useful in industrial applications, particularly the *baijiu* industry (Zheng et al., 2011; Jin et al., 2017; Ibarruri and Hernández, 2018). In a *Huaxi Xiaoqu* collected from Sichuan, China, the fungal community was relatively simple, consisting only of a few species, in which *Rhizopus stolonifera* constituted 94.98% of the community (Wu et al., 2017). Wang B. et al. (2018) found that the *Rhizopus* was the most abundant genus (above 60.00%) represented in all six *Jiuqu* samples, where its secreted glucoamylase and played a vital role as a saccharifying agent. In *baijiu* fermentation, alpha-amylase and glucoamylase were related to starch hydrolysis and affiliated with *Rhizopus*, *Aspergillus*, and *Rhizomucor* (Wang et al., 2020). In addition, *Rhizopus* can produce lactic acid in high quantities with potential applications in the production of rice-flavor *baijiu* (Yin et al., 2020). In addition to *Rhizopus*, yeasts, including *Candida* and *Pichia*, were important non-*Saccharomyces* yeasts in the saccharification process. Yeasts are the most important fungal populations contributing to *baijiu* quality in the solid-state fermentation process (Wu et al., 2013). Traditionally, *Xiaoqu* is usually prepared by cultivating mold in rice flour and, in some cases, with yeast (Yin et al., 2020). Although the starter selected in this study is *Rhizopus xiaoqu*, we identified a rich population of yeast in the initial saccharification stage by combining the results of culture-dependent and -independent analyses. Pang et al. (2020) reported that *Pichia* and *Candida* were the main fungal genera at the end of soaking, positively correlated with most flavors. Interestingly, the lower abundances of *Pichia*, *Wickerhamomyces*, and *Aspergillus* in fermented grain might decrease flavor compounds, including volatile alcohols, aromatics, and esters (Wang X. et al., 2018). Also, members of *Pichia* are good ethanol producers identified in *baijiu* fermentation (Jin et al., 2017; Li et al., 2018). In the light-flavor *baijiu* fermentation process, the genus *Candida* was positively correlated with acetic acid (Wang X. et al., 2018). This appears to be evidence that these fungi subtly shape the flavor quality of *baijiu*.

To explore the contribution of each operation in the pretreatment stage to the microbiota formation of the saccharification process, we analyzed the shared microorganisms at all sampling points via a petal diagram and revealed the potential connections regarding microbial composition between the two processes with the help of Sourcetracker. Most microorganisms in raw materials were killed after the steaming treatment (Pang et al., 2020). Considering that cooling processing was performed in an open environment, the microbiota in cooled samples was presumably derived from the environment. Environmental microbiota is an important source of fermentation microbiota and could drive both microbial succession and metabolic profiles during *baijiu* fermentation (Wang X. et al., 2018). In this study, the microorganisms inhabiting raw materials after cooling, *Qu*, and RH were closely related to the microbiota involved in the saccharification process. Similarly, in terms of the fermentation of light-flavor *baijiu*, sorghum after cooling and *Daqu* contributed 51.4% and 23.9% to the bacterial community in fermented grains (Pang et al., 2020). *Daqu* was also considered the main source of the fungal community in fermented grains (Wang X. et al., 2018).

Furthermore, based on the KEGG pathway, PICRUSt2 analyzed the sample’s potential functional characteristics to explain changes in microbial metabolic functions during two processes. Glucoamylase and alpha-1,4-glucan phosphorylase were the main enzymes attributed to the genus *Rhizopus* in *Jiuqu* (Wang B. et al., 2018). Wang et al. (2020) reported that alpha-amylase and glucoamylase are positively related to starch hydrolysis and ethanol production. They also indicated that these key saccharifying enzymes are associated with alcoholic fermentation in *baijiu* fermentation. In contrast, the bacterial community exhibited a greater contribution to alcohol dehydrogenase (EC:1.1.1.1), essential for ethanol production during the production of fermented foods. Huang et al. (2020) found that *Lactobacillus acetotolerans* strongly contributed to alcohol dehydrogenase, and it positively correlates with the content of ethanol in the fermented grains. L-lactate dehydrogenase (EC:1.1.1.27) is required for lactate and acetate production. The gene abundance for this enzyme in the P5_CO sample’s bacterial community was highest and had a higher abundance in saccharification’s middle and late stages. LAB, including *Lactobacillus plantarum*, *Lactobacillus acetotolerans*, and *Lactobacillus brevis* are essential contributors to L-lactate dehydrogenase (Huang et al., 2020). In addition, specific sugars can promote the interactions of the core microbial community members and ethanol production in a simulative *baijiu* fermentation under laboratory, which are associated with the saccharifying enzymes from starters (Wang et al., 2021). In general, the saccharification process’s fungal community is shaped by *Qu* and exhibited higher functional enzyme activity. Moreover, the activity of these enzymes in the bacterial community could contribute to flavor compounds’ metabolism.

## Conclusion

In summary, this study enhances our understanding of the relationship of the pretreatment and saccharification phase microbiota. Mainly the cooling raw material’s and RH’s bacterial community and *Qu’s* fungal community exhibited an important contribution in shaping the microbiota of the saccharification process. *Lactobacillus* and *Weissella* were the dominant bacteria in the P5_CO and RH samples, and the *Rhizopus* genus was the most dominant fungi in the *Qu* sample; these microbes were leading contributors of functional enzymes throughout saccharification. Further research will explore the contribution of the microbiota inhabiting the pretreatment and saccharification phases to the formation and accumulation of *fuyu*-flavor *baijiu* flavors and provide a strategy for better management of the traditional solid-state fermentation process.

## Data Availability Statement

The datasets presented in this study can be found in online repositories. The names of the repository/repositories and accession number(s) can be found in the article/Supplementary Material.

## Author Contributions

JK and YH analyzed the data and wrote the manuscript. JK, XZ, and HL performed the experiments. BH and XZ proofread the revised manuscript. ZD, LY, JC, LF, HZ, and XZ provided experimental samples and financial support of this study. All authors contributed to manuscript revision and approved the submitted version.

## Conflict of Interest

ZD, HL, and XZ were employed by the company Nutrition & Health Research Institute, COFCO Corporation. LY, JC, LF, HZ, and XZ were all employed by Jiugui Liquor Co., Ltd. The remaining authors declare that the research was conducted in the absence of any commercial or financial relationships that could be construed as a potential conflict of interest.

## Publisher’s Note

All claims expressed in this article are solely those of the authors and do not necessarily represent those of their affiliated organizations, or those of the publisher, the editors and the reviewers. Any product that may be evaluated in this article, or claim that may be made by its manufacturer, is not guaranteed or endorsed by the publisher.

## Footnotes

1
http://www.arb-silva.de

2
https://unite.ut.ee/
