# Supplementary material for: Deciphering the Shifts in Microbial Community Diversity From Material Pretreatment to Saccharification Process of Fuyu-Flavor Baijiu
Source: Front Microbiol. 2021 Aug 20;12:705967. doi: 10.3389/fmicb.2021.705967 (PMC8417803; doi:10.3389/fmicb.2021.705967)
Supplement: Supplementary file 1 [file Data_Sheet_1.docx]

Supplementary Material

**Table 1** Bacterial composition of initial saccharified samples isolates based on partial 16S gene sequencing identification.

| **Genus** | **Strain** | **Species identification** | **Related Genbank sequence** | **Identify (%)** |
| --- | --- | --- | --- | --- |
| ***Acetobacter*** | JGB14 | *Acetobacter indonesiensis* | LC379065 | 100 |
|  | JGB27 | *Acetobacter malorum* | MK583489 | 100 |
| ***Acinetobacter*** | JGB16 | *Acinetobacter lwoffii* | KF818633 | 100 |
| ***Bacillus*** | JGB5 | *Bacillus velezensis* | MK130896 | 100 |
|  | JGB6 | *Bacillus vallismortis* | MG836669 | 100 |
|  | JGB7 | *Bacillus velezensis* | MH000677 | 100 |
|  | JGB8 | *Bacillus velezensis* | MK367782 | 98 |
|  | JGB11 | *Bacillus amyloliquefaciens* | MT081100 | 100 |
|  | JGB13 | *Bacillus subtilis* | HQ441254 | 100 |
|  | JGB22 | *Bacillus siamensis* | MT672502 | 99 |
| ***Franconibacter*** | JGB20 | *Franconibacter pulveris* | MG705550 | 98 |
| ***Lactobacillus*** | JGB10 | *Lactobacillus hilgardii* | EU789397 | 99 |
|  | JGB25 | *Lactobacillus fermentum* | MT463847 | 100 |
|  | JGB26 | *Lactobacillus paracasei* | MT545150 | 100 |
|  | JGB28 | *Lactobacillus plantarum* | MT510499 | 100 |
|  | JGB29 | *Lacticaseibacillus paracasei* | MW866633 | 98 |
|  | JGB30 | *Lactobacillus paracasei* | MT545150 | 100 |
| ***Lactococcus*** | JGB17 | *Lactococcus lactis* | MT473312 | 100 |
|  | JGB19 | *Lactococcus lactis* subsp. *hordniae* | MT416439 | 98 |
|  | JGB21 | *Lactococcus taiwanensis* | MT573703 | 100 |
| ***Leuconostoc*** | JGB2 | *Leuconostoc lactis* | MT604792 | 99 |
|  | JGB4 | *Leuconostoc lactis* | MK100446 | 100 |
|  | JGB15 | *Leuconostoc lactis* | MT545082 | 100 |
|  | JGB24 | *Leuconostoc pseudomesenteroides* | MG550992 | 100 |
| ***Pantoea*** | JGB9 | *Pantoea* sp. | MH782068 | 99 |
| ***Paraburkholderia*** | JGB1 | *Paraburkholderia fungorum* | MG576015 | 98 |
| ***Staphylococcus*** | JGB23 | *Staphylococcus* sp. | MH071157 | 100 |
| ***Weissella*** | JGB3 | *Weissella confusa* | MT613537 | 99 |
|  | JGB12 | *Weissella cibaria* | MT463598 | 100 |
|  | JGB18 | *Weissella confusa* | MT515858 | 100 |

**Table 2** Fungal composition of initial saccharified samples isolates based on partial ITS gene sequencing identification.

| **Genus** | **Strain** | **Species identification** | **Related Genbank sequence** | **Identify (%)** |
| --- | --- | --- | --- | --- |
| ***Candida*** | JGF3 | *Candida akabanensis* | MT102815 | 99 |
|  | JGF5 | *Candida inconspicua* | EU315757 | 100 |
|  | JGF7 | *Candida inconspicua* | EU315757 | 100 |
|  | JGF10 | *Candida inconspicua* | EU315757 | 99 |
|  | JGF19 | *Candida tropicalis* | KP675692 | 99 |
|  | JGF27 | *Candida inconspicua* | EU315757 | 99 |
| ***Debaryomyces*** | JGF25 | *Debaryomyces hansenii* | MW710853 | 100 |
| ***Diutina*** | JGF14 | *Diutina rugosa* | KY985268 | 99 |
| ***Hyphopichia*** | JGF15 | *Hyphopichia burtonii* | KY103597 | 100 |
| ***Issatchenkia*** | JGF13 | *Issatchenkia orientalis* | AB467299 | 100 |
|  | JGF26 | *Issatchenkia orientalis* | AB467299 | 100 |
| ***Pichia*** | JGF1 | *Pichia anomala* | FJ713067 | 100 |
|  | JGF2 | *Pichia kudriavzevii* | MH545928 | 100 |
|  | JGF6 | *Pichia kudriavzevii* | KP674839 | 100 |
|  | JGF9 | *Pichia manshurica* | KP674783 | 100 |
|  | JGF16 | *Pichia kudriavzevii* | MT321167 | 100 |
|  | JGF17 | *Pichia kudriavzevii* | JF896573 | 100 |
|  | JGF20 | *Pichia kudriavzevii* | MT321167 | 100 |
|  | JGF22 | *Pichia kudriavzevii* | KP674839 | 100 |
|  | JGF29 | *Pichia kudriavzevii* | MT321167 | 100 |
| ***Saccharomyces*** | JGF8 | *Saccharomyces cerevisiae* | KY711301 | 97 |
|  | JGF11 | *Saccharomyces cerevisiae* | KY711301 | 98 |
|  | JGF21 | *Saccharomyces cerevisiae* | KC621078 | 98 |
|  | JGF23 | *Saccharomyces cerevisiae* | KC544490 | 98 |
|  | JGF28 | *Saccharomyces cerevisiae* | KY711301 | 98 |
| ***Wickerhamomyces*** | JGF4 | *Wickerhamomyces anomalus* | MH545921 | 100 |
|  | JGF12 | *Wickerhamomyces anomalus* | KY209903 | 99 |
|  | JGF18 | *Wickerhamomyces anomalus* | MH545921 | 100 |
|  | JGF24 | *Wickerhamomyces anomalus* | FN393999 | 100 |
|  | JGF30 | *Wickerhamomyces anomalus* | FN393999 | 100 |
